# Short and long-term costs among women experiencing preterm labour or preterm birth: the German experience

**DOI:** 10.1186/s12884-018-1912-0

**Published:** 2018-07-04

**Authors:** Shibani Pokras, Jeanne Pimenta, Evie Merinopoulou, Dimitra Lambrelli

**Affiliations:** 1 Value Evidence & Outcomes, GlaxoSmithKline, 1250 South Collegeville Road, Upper Providence, PA, 19426 USA; 2 Real World Evidence and Epidemiology, GlaxoSmithKline, London, UK; 3 Real World Evidence, Evidera, London, UK

**Keywords:** Premature birth, Obstetric labour, premature, Cohort studies, Cost and cost analyses, Germany

## Abstract

**Background:**

Preterm labour and birth (PTL/PTB) is characterised by major health and developmental risks for children, life–changing consequences for their families, and substantial healthcare and economic challenges for wider society. While it is known that PTL/PTB impacts infant healthcare costs in the short and long term in Germany, maternal costs have not been described in detail. The aim of this study was to comprehensively describe costs and resource use among PTL/PTB mothers during pregnancy, at hospitalisation for delivery, and up to three years after delivery—overall and according to gestational age (GA) at delivery.

**Methods:**

This study used data from the Statutory Health Insurance (SHI) sample of the AOK Hessen database in Germany. Mothers aged 12–44 years with deliveries between 2009 and 2013 and > 9 months of medical history prior to delivery were included. PTL/PTB mothers were defined by an International Classification of Diseases, 10th Revision (ICD-10) code for PTL during pregnancy, a diagnosis-related group (DRG) code indicating birthweight < 2500 g, or delivery of an infant < 37 weeks GA. Inpatient and outpatient resource use and total direct medical costs were examined during pregnancy, at delivery hospitalisation, and up to three years post-delivery.

**Results:**

Of all mothers, 2147 (20%) experienced PTL/PTB. During pregnancy, median costs for PTL/PTB mothers were €2130. During delivery hospitalisation, the mean length of stay for all PTL/PTB mothers was 6.0 days, and median costs were €2037. Length of stay and costs declined with increasing GA. Long term, PTL/PTB mothers’ total median costs were €607 in Year 1, €332 in Year 2, and €388 in Year 3 post-delivery. In each year after delivery, median costs appeared to be greater for mothers who delivered at lower GAs.

**Conclusion:**

In this description of costs and resource use among PTL/PTB mothers in Germany throughout the pregnancy and up to three years after delivery, the greatest costs were noted prior to delivery. Costs appeared to decrease with increasing GA, particularly during the delivery hospitalisation and the first year after delivery.

**Electronic supplementary material:**

The online version of this article (10.1186/s12884-018-1912-0) contains supplementary material, which is available to authorized users.

## Background

Preterm labour (PTL) is defined as regular uterine contractions, accompanied by cervical change, that occurs before 37 weeks gestation [1]. Approximately half of all spontaneous PTL cases result in preterm birth (PTB), [2] defined as childbirth at fewer than 37 completed weeks of gestation [3]. Although PTB can be a consequence of spontaneous PTL, it is possible for PTB to arise from other situations, such as if the mother has delivered following preterm premature rupture of membranes or underwent elective or iatrogenic preterm delivery [4]. In the United States (US), approximately 9.7% of births in 2015 were PTB, [5] and it has been estimated that PTL precedes approximately 50% of these [1]. In Europe, prevalence estimates of PTB in 2010 ranged from 4.1% (Belarus) to 14.7% (Cyprus) [3]. The estimated prevalence of PTB in Germany in 2010 was 9.2% [3].

PTB is a leading cause of neonatal morbidity and mortality [3]. PTB has been associated with a range of complications for the infant, including cerebral palsy, sensory deficits, learning disabilities, and respiratory illnesses, which persist into later life [6]. PTB also imposes a considerable burden on healthcare resources due to longer and more intensive hospital stays for the infant [7–10]. A study from the US found high annual costs associated with PTB infant hospitalisations and re-hospitalisations, totalling $13 billion in 2009, [11] and costs for PTB infants in several other countries have been shown to be significantly higher compared with term infants [8, 12, 13]. There is strong evidence that infant costs and outcomes vary according to gestational age (GA). Previous studies have shown a decrease in neonatal morbidity with each week of increase in GA, and delaying delivery even by one or two weeks can impact morbidities as well as costs [13–16].

The impact of PTL/PTB on maternal outcomes, resource use, and costs is less well described. In a US study, pregnancies with a PTL admission were shown to lead to significantly poorer outcomes compared with pregnancies without a PTL admission, with higher rates of maternal intensive care unit (ICU) admission, inpatient maternal mortality, and 30-day maternal mortality [17]. In a study in the Netherlands, mothers who experienced spontaneous PTL had significantly higher number of hospitalisations during pregnancy, with longer visits and more days spent in hospital during delivery [18]. The Institute of Medicine has reported that the projected maternal delivery costs of all women with PTB in the US (12.5% of all births) amounted to $1.9 billion in 2007 (corresponding average maternal delivery costs were estimated as $3800 per infant born) [19]. The long-term consequences of PTL/PTB among mothers are poorly described, despite the potential impact of PTL/PTB on direct and indirect longer-term cost. Giving birth to a preterm infant has been associated with poor maternal mental health, [20, 21] which may result in further healthcare interactions, and, as PTB is associated with a higher rate of disabilities in infants, [22] may increase caring responsibilities and cause increases in time off work. Despite this, there are very few studies investigating the impact of PTB on long-term maternal resource use and costs, and these are commonly not assessed in burden of illness studies in PTL/PTB.

The aim of this analysis was to describe resource use and costs among PTL/PTB mothers during pregnancy, during delivery hospitalisation, and up to three years after delivery—overall and stratified according to GA of infants at delivery—using data from a German health insurance fund. Our analysis was descriptive in nature, and aimed to estimate the absolute rather than excess costs incurred by PTL/PTB mothers.

## Methods

### Data source

This study utilised administrative insurance claims data from the Statutory Health Insurance (SHI) sample of AOK Hessen (Versichertenstichprobe AOK Hessen/KV Hessen) [23]. Hessen is a state in central Germany that includes the major cities of Frankfurt and Wiesbaden. The population of the state was estimated at six million individuals in 2012; of these, 1.5 million were insured by AOK. The sample available for research (SHI) is acquired by drawing a random sample of individuals insured by the AOK with a constant selection set of 18.8%. The current SHI sample used in this study included 353,284 persons who were insured in AOK Hessen for at least one day during the five-year period of 2009–2013. The sample is population-based without disease-related selection, with no disease-related dropouts, no recall bias, and a high level of data reliability; this enables patient-based observation and a bottom-up approach to disease costing from the perspective of the health insurance fund. The SHI dataset contains details on healthcare transactions related to insured persons and healthcare providers, including data on care received in general practice, outpatient care (all specialist visits), and hospital care, including emergency visits. Details of this database have been previously published [23, 24].

### Study population

We included mothers in the SHI sample who had a recorded diagnosis-related group (DRG) delivery code and a German procedure classification (Operationen- und Prozedurenschlüssel [OPS]) delivery code (Additional file 1) in the relevant study period (1 January 2009—31 December 2013). We further required women to be aged ≥ 12 and < 45 years at delivery and to have at least nine months of medical history available. We excluded women with more than one DRG delivery code within four months, those with no definite date of delivery, and those with a delivery discharge date in 2014. The index date was defined as the delivery date in the eligibility period. Women with multiple pregnancies during the study eligibility period were included in the study cohort once for each delivery, meaning that one woman could appear multiple times within the dataset. The baseline period was defined as the nine months preceding the index date. The delivery hospitalisation for mothers started from the day of hospital admission until the day of hospital discharge. Follow-up started from delivery hospitalisation discharge and lasted until the last date of data collection (31 December 2013), transfer out of the insurance fund, or the death of the mother, whichever occurred first. For women with multiple pregnancies, follow-up after each pregnancy lasted from hospital discharge until the beginning of the next pregnancy (defined as nine months [280 days] before the delivery date of the consequent pregnancy).

A cohort of PTL/PTB mothers was identified using any of the following criteria:

- An International Classification of Diseases, 10th Revision (ICD-10) code indicating PTL during pregnancy and/or PTB (Additional file 2)
- An estimated difference between the date of conception (calculated by the expected date of delivery) and the actual delivery date (defined by OPS codes) of less than 37 weeks
- A DRG code indicating infant’s birthweight < 2500 g (Additional file 2)
- For the subset of women who could be linked to their infant, delivery of a preterm infant using ICD-10 codes from infant records was also used to classify a mother as PTL/PTB.^1^

It should be noted that not all mothers with PTL delivered a preterm infant, and delivery of a preterm infant was not a condition for being included in the study. This is because not all cases of PTL necessarily result in PTB [2].

Mothers who did not meet the criteria for PTL/PTB were considered non-PTL/PTB mothers. GA was defined using a recorded variable available within the database indicating the expected date of delivery. To calculate the GA, we used this expected date of delivery to calculate an estimated date of conception. This was done by assuming that all pregnancies’ estimated delivery date had been estimated as 280 days after the date of conception. By subtracting 280 days from the expected date of delivery, we derived an estimated date of conception. The difference between this estimated conception date and the actual delivery date was the GA. Additionally, ICD-10 codes present in the mother’s record during birth (P07.2 [extreme immaturity, GA < 28 weeks] and P07.3 [other preterm infants, GA 28–36 weeks] and O09 [duration of pregnancy]) were used to define GA. Mothers with missing GA were assigned to the > 37 weeks of GA based on the distribution of GA in the rest of the population.

Driven by the GA groups, as defined by ICD-10 codes, mothers were subsequently classified into three groups based on their infant's GA:

- < 28 weeks (extremely preterm infants)
- 28–36 weeks (other preterm infants)
- ≥37 weeks (term infants)

### Study measures

#### Maternal characteristics

Data on demographics and clinical characteristics were assessed at delivery and during the nine-month baseline period. Characteristics of interest included age at delivery, plurality of births (multiple or singleton), infant GA, and maternal risk factors for PTL, [25] which could be captured in the AOK database through ICD-10 diagnosis codes: hypertension, diabetes mellitus, gestational diabetes, and depression. Information on baseline clinical conditions was used to calculate the updated Charlson Comorbidity Index (CCI) to estimate the overall health status of the mothers [26]. We used diagnosis codes recorded in the inpatient and outpatient setting to define the presence of clinical conditions and maternal risk factors for PTL.

#### Resource use and costs

Resource use and total direct medical costs (in Euros) were examined during pregnancy, at delivery hospitalisation, and up to three years post-delivery. During each period, outpatient resource use, outpatient prescription data, inpatient resource use, and other services (defined below), which included all services not reimbursed in the inpatient or outpatient setting, were considered. Specifically, we considered the following resources in the outpatient setting: laboratory tests, preventative procedures (such as cancer screening and vaccinations), basic procedures (such as ultrasounds, magnetic resonance imaging [MRI], or echocardiograms [ECG]), prescribed medications, general practitioner [GP] visits, gynaecologist/paediatrician visits, and any other specialist visit. It should be noted that due to the nature of the reimbursement system in Germany, which reimburses outpatient physicians on a quarterly basis, visits to physicians are recorded as one per quarter irrespective of how many encounters took place within the same quarter. Other services considered were: remedies (such as massages or occupational therapy), medical devices, midwifery services, driving services, and other (such as household help or home care). Outpatient costs were estimated as total costs for each service used or drug prescribed. In the inpatient setting we considered the total number of all-cause hospitalisations, length of stay (LOS), pregnancy/labour procedures, diagnostic tests, or therapeutic procedures (such as operations) performed during hospitalisation. Inpatient costs were estimated based on DRG codes per hospital stay. Costs were estimated from the third-party payer perspective, corresponding to the SHI fund, which is in accordance with Institute for Quality and Efficiency in Health Care (IQWiG) guidelines for cost of illness analyses in the German setting [27].

As not all mothers were insured for 365 days in the respective years of follow-up (lost to follow-up or reached the end of the observation period), costs for the first, second, and third year of follow-up were evaluated for those mothers who had sufficient follow-up and were continually insured in the respective year. It should be noted that we allowed women to enter the cohort up until the end of the study period (31 December 2013)—this means that only women enrolled prior to the 1 January 2011 could accrue the full three years of follow-up, and among these, only those who did not die or were lost-to-follow-up (LTFU) were observed for the full three years. We nonetheless chose to include women who could not be followed for the full three years to maximise the study cohort available for analysis.

### Statistical methods

Continuous variables were described using average values (median and mean) and measures of data dispersion (interquartile range [IQR], minimum and maximum values). Categorical variables were described using frequencies and percentages. *P*-values comparing the characteristics of PTL/PTB mothers to non-PTL/PTB mothers were calculated using the chi-squared test/Fisher’s Exact test in those instances when expected cell counts < 5. Univariable negative binomial models were used to estimate the rate of resource utilisation during follow-up as the number of events per person-year with 95% confidence intervals (CI). Costs were presented using summary statistics, which were estimated and included mothers without any resource use (i.e., median and mean costs were estimated, including women who incurred zero costs).

All data programming and analyses were carried out using Microsoft SQL server 2008 (Microsoft, Redmond, WA, USA) and SAS for Windows Release 9.3 (SAS Institute Inc., Cary, NC, USA).

## Results

### Cases of PTL/PTB

**Fig. 1:**
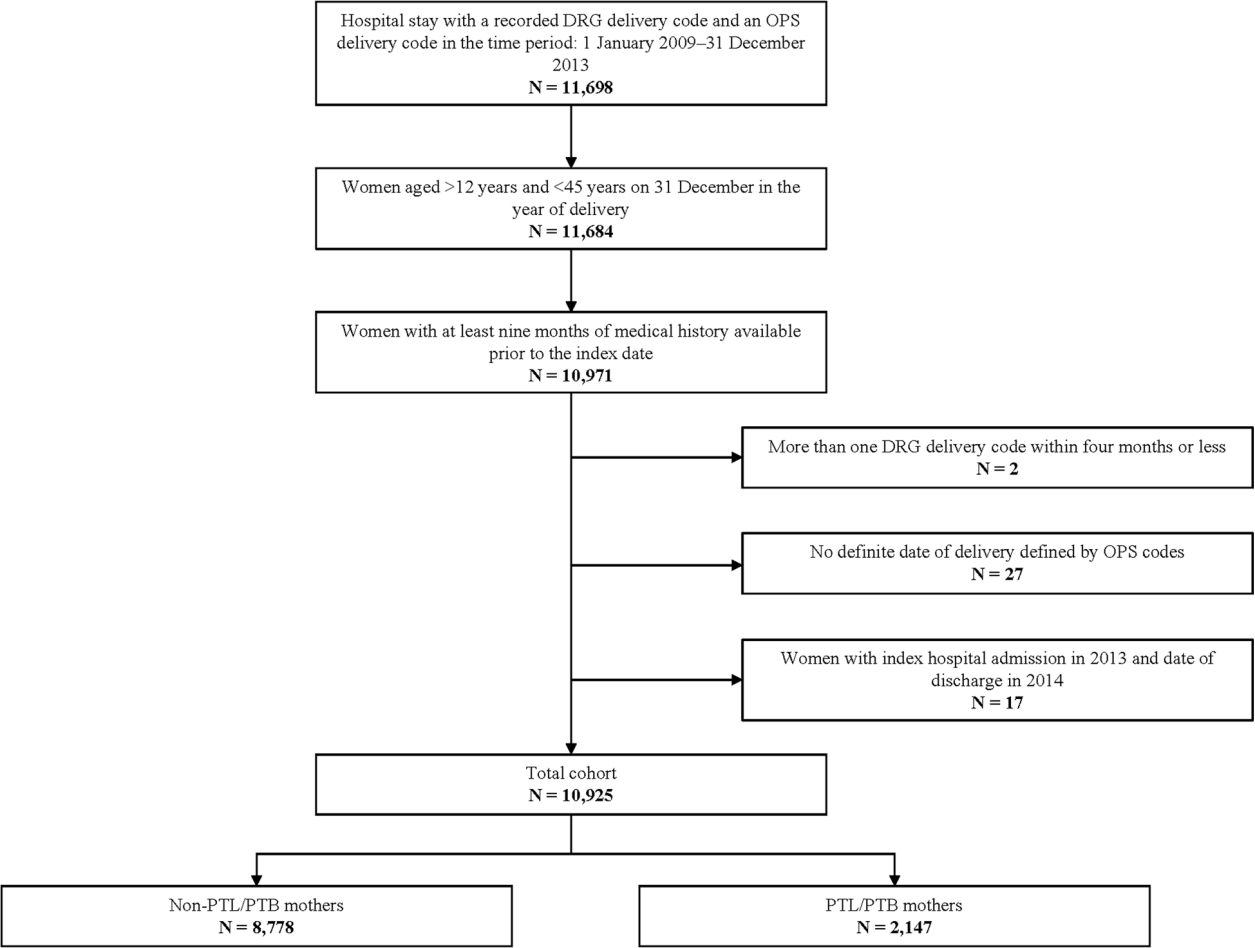
Flowchart of the Cohort Selection. Abbreviations: DRG = diagnosis-related group; PTB = preterm birth; PTL = preterm labour; OPS = Operationen- und Prozedurenschlüssel

In total, we identified 10,925 mothers in the SHI sample that were eligible for inclusion. An illustration of the cohort selection process can be seen in Fig. 1. Among these 10,925 mothers, 97.7% gave birth to singletons (*N* = 10,678) and 2.3% to multiples (*N* = 247). Approximately 19.7% (*N* = 2147) of the mothers had a diagnosis of PTL during pregnancy or had a PTB. Most mothers (88.2%) had an expected date of delivery variable, which allowed us to directly calculate the GA. A further 1.7% could be categorised into a GA group based on ICD-10 codes, and the remaining 10.1% were assigned to the ≥ 37 weeks GA group as described in the methods section. All mothers who were identified based on giving birth to a low-birth-weight (LBW) infant also had an estimated GA indicating a PTB. Overall, most PTL/PTB mothers delivered at term (37 weeks GA or higher; 65.6%). Approximately 32.3% delivered between 28 and 36 weeks GA, and 2.1% delivered before 28 weeks GA. The proportion of PTL/PTB mothers who delivered at term differed depending on whether the mother gave birth to singletons or multiples (86.4% term for singletons; 41% for multiples).

### Maternal characteristics

The characteristics of PTL/PTB mothers are described in Table 1. Among the cohort of PTL/PTB mothers, most were aged between 19 and 30 years at delivery (59.4%), with an even distribution of births by calendar year. Most mothers had not given birth before (63.3%), and 41% delivered by Caesarean section. Approximately 11.0% had gestational diabetes, 4.3% had hypertension, and 2.3% had diabetes mellitus diagnosed during pregnancy and/or at delivery hospitalisation.

**Table 1:** Characteristics of PTL/PTB Mothers Compared with Non-PTL/PTB Mothers at Delivery

	PTL/PTB mothers	Non-PTL/PTB mothers	*P* –value^a^
	N = 2147	*N* = 8778	
	*N* (%)	*N* (%)	
Year of delivery			0.3403
2009	395 (18.4)	1645 (18.7)	
2010	420 (19.6)	1726 (19.7)	
2011	413 (19.2)	1819 (20.7)	
2012	472 (22.0)	1778 (20.3)	
2013	447 (20.8)	1810 (20.6)	
Maternal age at delivery^b^			< 0.0001
12–18 years	48 (2.2)	95 (1.1)	
19–30 years	1276 (59.4)	5194 (59.2)	
31–35 years	496 (23.1)	2232 (25.4)	
36–44 years	327 (15.2)	1257 (14.3)	
Nulliparous^c^	1360 (63.3)	5224 (59.5)	0.0011
Caesarean section	885 (41.2)	2769 (31.5)	< 0.0001
Birth plurality			< 0.0001
Singleton	1969 (91.7)	8709 (99.2)	
Twin	173 (8.1)	64 (0.0)	
Other multiple	5 (0.0)	3 (0.0)	
Charlson Comorbidity Index^d^			0.0142
0	1750 (81.5)	7332 (83.5)	
1	315 (14.7)	1155 (13.2)	
2	54 (2.5)	230 (2.6)	
3	21 (1.0)	41 (0.5)	
4+	7 (0.3)	20 (0.2)	
Selected comorbidities^d^			
Gestational diabetes	237 (11.0)	893 (10.2)	0.2378
Hypertension	93 (4.3)	243 (2.8)	0.0002
Depression	83 (3.9)	334 (3.8)	0.8950
Hypotension	66 (3.1)	268 (3.1)	0.9597
Diabetes mellitus	50 (2.3)	113 (1.3)	0.0004

^a^*P*-values compare PTL/PTB mothers with non-PTL/PTB mothers, and are calculated using the chi-squared test/Fisher’s Exact test in instances when expected cell counts < 5

^b^Values are rounded to the nearest whole number

^c^Refers to parity preceding index delivery

^d^Measured during the nine-month baseline period, diagnoses in inpatient or outpatient setting

Abbreviations: *PTB* preterm birth, *PTL* preterm labour

There were some significant differences between PTL/PTB mothers and non-PTL/PTB mothers (Table 1). Although most mothers were 19–30 years of age when giving birth, the proportion of mothers who were in the youngest (12–18 years of age) or oldest age categories (36–44 years of age) was greater among PTL/PTB mothers compared with non-PTL/PTB mothers (*p* < 0.0001). PTL/PTB mothers were also more likely to have a CCI score greater than 0 (*p* = 0.0142), as well as hypertension (*p* = 0.0002) and diabetes (*p* = 0.0004). Compared with non-PTL/PTB mothers, those with PTL/PTB were more likely to deliver by Caesarean section (*p* < 0.0001) and to give birth to twins (*p* < 0.0001).

### Resource use and cost during pregnancy

A breakdown of the frequency of resource use during pregnancy among PTL/PTB mothers can be seen in Table 2. Nearly all women utilised outpatient care during their pregnancy. Visits to the gynaecologist and other specialists were very common (85.8% and 93.8% of mothers, respectively), and almost half of women (48%) were hospitalised due to any cause at some point during their pregnancy. Most women received some form of medication during pregnancy (74%), with 7.1% of mothers receiving progesterone and 3.9% tocolytic drugs in the outpatient setting. Data on inpatient medication use was not available. A considerable proportion of women also utilised other care services, including midwifery services (53%), medical device use (25%), and driving services (e.g., ambulance or patient transport; 23%) during their pregnancy.

**Table 2:** Resource use and costs during pregnancy among PTL/PTB Mothers

	Resource use	Direct Medical Cost (Euros)	
	N with at least one use (%)	Mean	Median (IQR)
Outpatient care		1066	984 (752–1274)
GP visit (in at least one quarter)	1330 (62.0)	75	35 (0**–**106)
Gynaecologist visit (in at least one quarter)	1841 (85.8)	616	619 (409**–**815)
Specialist^a^ visit (in at least one quarter)	2014 (93.8)	375	188 (96**–**480)
Laboratory test	2125 (99.0)	61	34 (14**–**73)
Diagnostic test	1649 (76.8)	45	17 (3**–**48)
Pregnancy/labour procedure	2131 (99.3)	675	638 (501**–**818)
Therapeutic procedure^b^	924 (43.0)	41	0 (0**–**15)
Preventive procedure^b^	2103 (98.0)	24	30 (7**–**33)
Basic procedure^b^	2136 (99.5)	221	183 (110**–**285)
Prescribed medications	1590 (74.1)	188	36 (12**–**107)
Inpatient care^c^		1084	0 (0–1483)
All-cause hospitalisation	1030 (48.0)		
Pregnancy procedure	9 (0.4)		
Diagnostic test	62 (2.9)		
Therapeutic procedure	115 (5.4)		
Other services^d^		587	398 (42–817)
Remedies	99 (4.6)	6	0 (0**–**0)
Medical devices	537 (25.0)	29	0 (0**–**0)
Midwife services	1126 (52.5)	335	43 (0**–**540)
Driving services	482 (22.5)	128	0 (0**–**0)
Other	575 (26.8)	89	0 (0**–**16)
Total Costs		2925	2130 (1280–3594)

Including zero costs (insurant without resource)

^a^Specialist visit is defined as any physician group excluding GP and gynaecologist

^b^Examples of therapeutic procedures: Infusion, substitute assisted treatment for opiate addicts, verbal intervention in psychosomatic disease states; examples of preventative procedures: basic services for specialists, cytological examination (cancer screening), examination for early detection of cancer in women; examples of basic procedures include: personal physician-patient contact, shipping for materials, transport and transfer results, charge for dispatch of transport letters/ written materials

^c^Costs for each individual resource was not available, as costs were estimated based on DRG codes

^d^Note that other services include: Remedies (e.g., physiotherapy, massage, manual therapy, occupational therapy); Medical devices (e.g., measurement devices, medical devices, inhalation devices); Midwife services (e.g., antenatal preparation, home visits after birth); Driving services (e.g., ambulance service, patient transportation); Other (e.g., household help, home care necessary for medical treatment)

Abbreviations: *GP* general practitioner; *IQR* interquartile range, *PTB* preterm birth, *PTL* preterm labour

The median total cost among PTL/PTB mothers during pregnancy was €2130 (mean: €2925). Costs per sector and resource use are presented in Table 2. The highest median costs were observed in the outpatient sector (median: €984; IQR: €752–€1274; mean: €1066), whereas the highest mean costs were observed in the inpatient sector (€1084). As most women were not hospitalised during pregnancy, median costs in the inpatient sector were €0 (IQR: €0–€1483).

### Resource use and cost during delivery hospitalisation

**Fig. 2:**
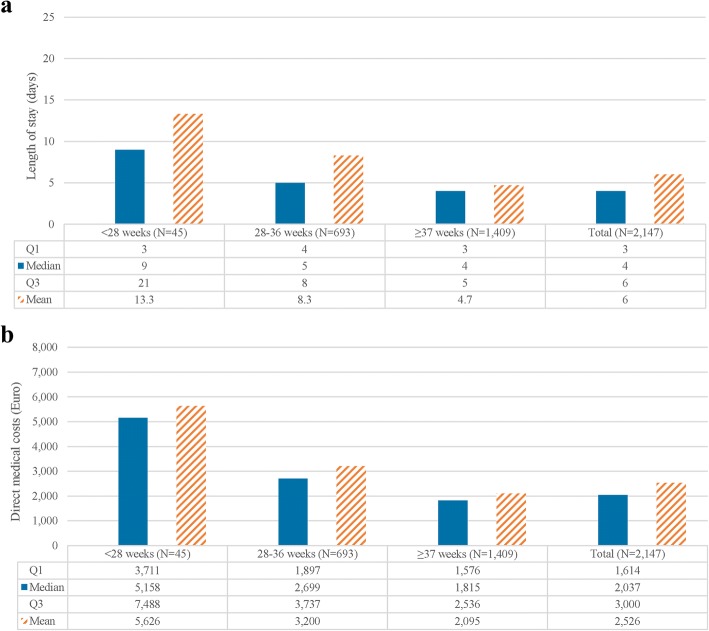
Resource use associated with the delivery hospitalisation among PTL/PTB mothers, by GA. **a** Length of stay (days) during delivery hospitalisation. **b** Direct medical costs in Euros during delivery hospitalisation

During delivery hospitalisation, the mean LOS for PTL/PTB mothers was 6.0 days (median: 4.0; IQR: 3.0–6.0). Mean LOS declined with increasing gestational age, from 13.3 days for < 28 weeks to 8.3 days for 28–36 weeks and 4.7 days for ≥37 weeks (Fig. 2a). Trends were similar when considering median LOS.

Median total costs during delivery hospitalisation were €2037 (mean: €2525.8; IQR: €1614–€3000). Stratified by GA, costs declined with increasing GA; while the cost associated with delivery hospitalisation was €5158 (IQR: €3711–€7488) among mothers delivering with a GA of < 28 weeks, it declined to €2699 for mothers delivering at a GA of 28–36 weeks and €1815 (IQR: €1576–€2536) for mothers giving birth at ≥37 weeks (Fig. 2b). Trends were similar when considering mean costs (Fig. 2b).

Overall, the most common inpatient service used was ‘monitoring and management of a high-risk delivery’ (31%) followed by ‘monitoring and management of a normal birth’ (22%). However, this varied according to the GA. In the < 28 weeks GA group, the most common procedures were Caesarean section (33%) and post-partum instrumental removal of the placenta (16%). In contrast, the most common procedure in the ≥37 weeks group was episiotomy (13%).

### Long-term resource use and costs after delivery

The rate of resource use after delivery was found to decline with time since delivery for several of the resources considered (Tables 3, 4, and 5). Notably, in the outpatient sector, we observed a decline over time for the rate of quarters with gynaecological visits, which declined from 1.93 (95% CI: 1.87–1.99) quarters with visits per year during the first year after delivery to 0.93 (95% CI: 0.86–1.00) during the second year and 0.76 (95% CI: 0.68–0.84) during the third year. The rate of use of pregnancy and labour procedures in the outpatient sector also decreased, from 1.55 (95% CI: 1.49–1.61) uses per year in the first year to 0.37 (95% CI: 0.27–0.51) and 0.44 (95% CI:0.29–0.66) in the second and third year, respectively. It should be noted that the rate of use of some inpatient resources and other services was low across all years, making it hard to draw any conclusions regarding trends (Tables 3, 4 and 5).

**Table 3:** Resource use in first year post-discharge from delivery visit hospitalisation among PTL/PTB Mothers, by GA

	First year post discharge			
	< 28 weeks	28–36 weeks	≥37 weeks	All
	Rate (95% CI)	Rate (95% CI)	Rate (95% CI)	Rate (95% CI)
Outpatient care				
Quarters with a GP visit	1.5 (1.1–2.1)	1.5 (1.4–1.6)	1.3 (1.2–1.4)	1.4 (1.3–1.5)
Quarters with a gynaecological visit	2.1 (1.7–2.5)	1.9 (1.8–2.0)	2.0 (1.9–2.0)	1.9 (1.9–2.0)
Quarters with a specialist visit	2.6 (2.2–3.1)	2.5 (2.4–2.6)	2.4 (2.3–2.5)	2.4 (2.4–2.5)
Pregnancy/labour procedures^a^	1.5 (1.1–2.0)	1.5 (1.4–1.6)	1.6 (1.5–1.6)	1.6 (1.5–1.6)
Laboratory tests	16.2 (10.5–25.1)	10.0 (8.9–11.2)	7.1 (6.6–7.7)	8.2 (7.7–8.8)
Diagnostic tests	5.6 (4.2–7.6)	3.6 (3.4–3.9)	3.4 (3.2–3.6)	3.5 (3.4–3.7)
Therapeutic procedures^b^	13.9 (6.2–31.2)	1.1 (1.0–1.4)	1.1 (0.9–1.2)	1.4 (1.2–1.5)
Preventative procedures	2.3 (1.8–3.0)	2.2 (2.0–2.3)	2.3 (2.2–2.5)	2.3 (2.2–2.4)
Basic procedures	16.4 (12.9–20.9)	12.3 (11.5–13.1)	11.3 (10.8–11.8)	11.7 (11.3–12.1)
Prescribed medications	5.6 (3.7–8.6)	3.2 (2.9–3.5)	2.3 (2.1–2.5)	2.6 (2.5–2.8)
Inpatient care				
All-cause hospitalisations	0.3 (0.2–0.5)	0.2 (0.1–0.2)	0.1 (0.1–0.2)	0.1 (0.1–0.2)
Pregnancy/labour procedures^c^	0.0 (0.0–0.2)	0.0 (0.0–0.1)	0.0 (0.0–0.0)	0.0 (0.0–0.0)
Diagnostic tests	0.2 (0.1–0.8)	0.0 (0.0–0.1)	0.1 (0.0–0.1)	0.1 (0.0–0.1)
Therapeutic procedures^d^	0.2 (0.1–0.5)	0.2 (0.1–0.3)	0.1 (0.1–0.2)	0.1 (0.1–0.2)
Other services^e^				
Remedy services	0.1 (0.0–0.6)	0.1 (0.1–0.2)	0.1 (0.1–0.1)	0.1 (0.1–0.1)
Medical device services	1.9 (1.2–3.1)	1.1 (1.0–1.2)	0.5 (0.4–0.5)	0.7 (0.6–0.8)
Midwife services	0.3 (0.1–0.6)	0.6 (0.6–0.7)	0.6 (0.6–0.6)	0.6 (0.6–0.6)
Driving services	0.3 (0.1–0.6)	0.1 (0.1–0.1)	0.1 (0.1–0.1)	0.1 (0.1–0.1)
Other	0.4 (0.2–0.8)	0.4 (0.3–0.4)	0.2 (0.2–0.3)	0.3 (0.2–0.3)

^a^Any physician group excluding general practitioner and gynaecologist

^b^Examples of outpatient therapeutic procedures: Infusion, substitute assisted treatment for opiate addicts, verbal intervention in psychosomatic disease states; examples of preventative procedures: basic services for specialists, cytological examination (cancer screening), examination for early detection of cancer in women; examples of basic procedures include: personal physician-patient contact, shipping for materials, transport and transfer results, charge for dispatch of transport letters/written materials

^c^This excluded subsequent pregnancies resulting in live births, but could include procedures such as abortions or procedures not resulting in live births

^d^Examples of inpatient therapeutic procedures include: reconstruction of cervix, preventative measures (e.g., education and basic training), monitoring (respiratory, cardiac, circulatory)

^e^Note that other services include: Remedies (e.g., physiotherapy, massage, manual therapy, occupational therapy); Medical devices (e.g., measurement devices, medical devices, inhalation devices); Midwife services (e.g., antenatal preparation, home visits after birth); Driving services (e.g., ambulance service, patient transportation); Other (e.g., household help, home care necessary for medical treatment)

Abbreviations: *CI* confidence interval, *GA* gestational age, *GP* general practitioner, *PTB* preterm birth, *PTL* preterm labour

**Table 4:** Resource use in second year post-discharge from delivery visit hospitalisation among PTL/PTB mothers, by GA

	Second year post discharge			
	< 28 weeks	28–36 weeks	≥37 weeks	All
	Rate (95% CI)	Rate (95% CI)	Rate (95% CI)	Rate (95% CI)
Outpatient care				
Quarters with a GP visit	1.0 (0.5–1.7)	1.4 (1.3–1.6)	1.3 (1.2–1.5)	1.4 (1.3–1.5)
Quarters with a gynaecological visit	0.7 (0.3–1.6)	1.0 (0.9–1.1)	0.9 (0.8–1.0)	0.9 (0.9–1.0)
Quarters with a specialist visit	2.4 (1.9–3.0)	2.1 (2.0–2.3)	2.0 (1.9–2.1)	2.1 (2.0–2.1)
Pregnancy/labour procedures^b^	0.4 (0.1–2.2)	0.3 (0.2–0.6)	0.4 (0.3–0.6)	0.4 (0.3–0.5)
Laboratory tests	11.4 (5.5–23.6)	9.4 (7.9–11.3)	7.0 (6.2–7.8)	7.9 (7.1–8.7)
Diagnostic tests	3.0 (1.8–5.2)	3.0 (2.7–3.5)	2.8 (2.5–3.0)	2.9 (2.7–3.1)
Therapeutic procedures^c^	7.7 (2.7–22.6)	1.6 (1.3–2.1)	1.2 (1.1–1.5)	1.5 (1.3–1.7)
Preventative procedures	1.7 (1.0–2.7)	1.8 (1.6–2.1)	1.7 (1.6–1.9)	1.8 (1.7–1.9)
Basic procedures	13.8 (9.7–19.6)	11.4 (10.3–12.6)	9.9 (9.3–10.7)	10.5 (9.9–11.1)
Prescribed medications	4.0 (2.5–6.4)	3.2 (2.8–3.7)	2.6 (2.4–2.9)	2.8 (2.6–3.1)
Inpatient care				
All-cause hospitalisations	0.3 (0.2–0.7)	0.2 (0.1–0.2)	0.1 (0.1–0.1)	0.1 (0.1–0.2)
Pregnancy/labour procedures^d^	a	0.0 (0.0–0.0)	0.0 (0.0–0.0)	0.0 (0.0–0.0)
Diagnostic tests	0.3 (0.1–1.2)	0.1 (0.0–0.2)	0.0 (0.0–0.1)	0.1 (0.0–0.1)
Therapeutic procedures^e^	0.1 (0.0–0.5)	0.1 (0.1–0.2)	0.1 (0.1–0.2)	0.1 (0.1–0.2)
Other services^f^				
Remedy services	0.2 (0.0–1.5)	0.1 (0.1–0.2)	0.1 (0.1–0.1)	0.1 (0.1–0.1)
Medical device services	0.1 (0.0–0.8)	0.2 (0.2–0.3)	0.1 (0.1–0.1)	0.2 (0.1–0.2)
Midwife services	a	0.1 (0.1–0.1)	0.0 (0.0–0.0)	0.1 (0.1–0.1)
Driving services	0.1 (0.0–0.8)	0.1 (0.1–0.1)	0.1 (0.0–0.1)	0.1 (0.1–0.1)
Other	0.1 (0.0–0.8)	0.2 (0.2–0.3)	0.2 (0.2–0.2)	0.2 (0.2–0.2)

^a^Model did not converge

^b^Any physician group excluding general practitioner and gynaecologist

^c^Examples of outpatient therapeutic procedures: Infusion, substitute assisted treatment for opiate addicts, verbal intervention in psychosomatic disease states; examples of preventative procedures: basic services for specialists, cytological examination (cancer screening), examination for early detection of cancer in women; examples of basic procedures include: personal physician-patient contact, shipping for materials, transport and transfer results, charge for dispatch of transport letters/written materials

^d^This excluded subsequent pregnancies resulting in live births, but could include procedures such as abortions or procedures not resulting in live births

^e^Examples of inpatient therapeutic procedures include: reconstruction of cervix, preventative measures (e.g., education and basic training), monitoring (respiratory, cardiac, circulatory)

^f^Note that other services include: Remedies (e.g., physiotherapy, massage, manual therapy, occupational therapy); Medical devices (e.g., measurement devices, medical devices, inhalation devices); Midwife services (e.g., antenatal preparation, home visits after birth); Driving services (e.g., ambulance service, patient transportation); Other (e.g., household help, home care necessary for medical treatment)

Abbreviations: CI = confidence interval; GA = gestational age; GP = general practitioner; PTB = preterm birth; PTL = preterm labour

**Table 5:** Resource use in third year post-discharge from delivery visit hospitalisation among PTL/PTB Mothers, by GA

	Third year post discharge			
	< 28 weeks	28–36 weeks	≥37 weeks	All
	Rate (95% CI)	Rate (95% CI)	Rate (95% CI)	Rate (95% CI)
Outpatient care				
Quarters with a GP visit	1.1 (0.6–2.1)	1.3 (1.2–1.6)	1.2 (1.1–1.3)	1.24 (1.13–1.36)
Quarters with a gynaecological visit	0.5 (0.2–1.5)	0.8 (0.7–1.0)	0.7 (0.6–0.8)	0.76 (0.68–0.84)
Quarters with a specialist visit	2.0 (1.4–2.9)	2.3 (2.1–2.4)	2.2 (2.1–2.3)	2.21 (2.11–2.31)
Pregnancy/labour procedures^b^	a	0.2 (0.1–0.6)	0.6 (0.4–0.9)	0.44 (0.29–0.66)
Laboratory tests	9.2 (3.1–27.3)	9.9 (7.9–12.3)	8.2 (7.0–9.5)	8.76 (7.74–9.92)
Diagnostic tests	2.2 (0.9–4.9)	3.3 (2.8–3.9)	2.9 (2.6–3.3)	3.05 (2.77–3.35)
Therapeutic procedures^c^	1.5 (0.6–3.9)	1.8 (1.4–2.3)	1.6 (1.3–2.0)	1.67 (1.42–1.96)
Preventative procedures	0.5 (0.1–2.0)	1.7 (1.5–2.0)	1.9 (1.7–2.1)	1.83 (1.68–1.99)
Basic procedures	13.5 (6.2–29.4)	11.9 (10.5–13.5)	11.2 (10.3–12.2)	11.46 (10.68–12.30)
Prescribed medications	2.9 (1.3–6.3)	3.5 (3.0–4.1)	2.8 (2.5–3.2)	3.02 (2.75–3.32)
Inpatient care				
All-cause hospitalisations	0.2 (0.1–1.0)	0.2 (0.1–0.3)	0.1 (0.1–0.2)	0.15 (0.12–0.19)
Pregnancy/labour procedures^d^	a	0.1 (0.0–0.2)	0.0 (0.0–0.0)	0.01 (0.00–0.03)
Diagnostic tests	0.2 (0.1–1.0)	0.1 (0.0–0.2)	0.0 (0.0–0.1)	0.06 (0.03–0.10)
Therapeutic procedures^e^	0.4 (0.1–2.3)	0.3 (0.2–0.7)	0.2 (0.1–0.3)	0.21 (0.13–0.34)
Other services^f^				
Remedy services	0.4 (0.1–1.3)	0.1 (0.1–0.2)	0.1 (0.1–0.2)	0.12 (0.09–0.15)
Medical device services	a	0.2 (0.1–0.4)	0.1 (0.1–0.2)	0.15 (0.11–0.20)
Midwife services	a	0.1 (0.1–0.2)	0.0 (0.0–0.0)	0.01 (0.00–0.02)
Driving services	0.1 (0.0–0.6)	0.1 (0.1–0.2)	0.1 (0.0–0.1)	0.07 (0.05–0.11)
Other	a	0.2 (0.1–0.4)	0.2 (0.1–0.2)	0.18 (0.14–0.24)

^a^Model did not converge

^b^Any physician group excluding general practitioner and gynaecologist

^c^Examples of outpatient therapeutic procedures: Infusion, substitute assisted treatment for opiate addicts, verbal intervention in psychosomatic disease states; examples of preventative procedures: basic services for specialists, cytological examination (cancer screening), examination for early detection of cancer in women; examples of basic procedures include: personal physician-patient contact, shipping for materials, transport and transfer results, charge for dispatch of transport letters/ written materials

^d^This excluded subsequent pregnancies resulting in live births, but could include procedures such as abortions or procedures not resulting in live births

^e^Examples of inpatient therapeutic procedures include: reconstruction of cervix, preventative measures (e.g., education and basic training), monitoring (respiratory, cardiac, circulatory)

^f^Note that other services include: Remedies (e.g., physiotherapy, massage, manual therapy, occupational therapy); Medical devices (e.g., measurement devices, medical devices, inhalation devices); Midwife services (e.g., antenatal preparation, home visits after birth); Driving services (e.g., ambulance service, patient transportation); Other (e.g., household help, home care necessary for medical treatment)

Abbreviations: *CI* confidence interval, *GA* gestational age, *GP* general practitioner, *PTB* preterm birth, *PTL* preterm labour

The rate of resource use across settings of care in the three years after delivery, split by GA, can be seen in Tables 3, 4 and 5. In the first year after delivery, the largest differences in the rate of resource use was observed for outpatient therapeutic procedures, which declined considerably with increasing GA, from 13.93 (95% CI: 6.22–31.23) uses per person year in the < 28 weeks GA group to 1.13 (95% CI: 0.95–1.35) and 1.07 (95% CI: 0.94–1.22) in the 28–36 weeks and ≥ 37 weeks GA groups, respectively. The rate of use of laboratory tests and basic procedures was also significantly higher in the lowest GA group (Table 3). These differences were less marked during the second and third year after delivery (Table 4 and 5). Although the rate of use of inpatient resources was low, it appeared that the rate of hospitalisations decreased with increasing GA, from 0.29 (95% CI: 0.16–0.53) hospitalisations per person year in the < 28 weeks GA group to 0.15 (95% CI: 0.12–0.20) and 0.13 (95% CI: 0.11–0.15) in the ≥ 37 weeks GA groups, respectively. In contrast to the outpatient resources discussed, these estimates were similar during the second (Table 4) and third (Table 5) years after delivery. However, it should be noted that due to the small number of events, the CIs surrounding these estimates were wide.

During the follow-up period, PTL/PTB mothers’ total median costs were €607 (mean: €1087; 1422 mothers contributing data) in the first year, €332 (mean: €912; 861 mothers contributing data) in the second year, and €388 (mean: €1120; 484 mothers contributing data) in the third year (Table 6). The highest median and mean costs were incurred in the outpatient setting during all three years of follow-up, although this varied by GA. For mothers in the < 28 weeks GA group, the mean, and therefore total, costs were highest in the inpatient sector during all three years after delivery compared with other GAs. In all three years after delivery, mean and median costs appeared to be higher for mothers who gave birth at the lowest GA (< 28 weeks; Table 6) compared with mothers who gave birth at a higher GA. This gradient was most marked during the first year after delivery when considering median costs, whereas the decline by GA appeared reasonably constant across all three years after delivery when considering mean costs.

**Table 6:** Long-term costs (Euros) after delivery among PTL/PTB mothers, by GA and sector

	< 28 weeks			28–36 weeks			≥ 37 weeks			All		
	N	Mean	Median (IQR)	N	Mean	Median (IQR)	N	Mean	Median (IQR)	N	Mean	Median (IQR)
First Year Post Discharge	30			463			929			1422		
Outpatient sector		674	368 (263**–**580)		394	285 (190**–**482)		351	281 (181**–**430)		372	283 (187–448)
Inpatient sector		815	0 (0**–**101)		313	0 (0**–**0)		236	0 (0**–**0)		273	0 (0**–**0)
Prescribed medications		312	121 (14**–**307)		219	39 (0**–**107)		93	27 (0**–**68)		139	30 (0**–**81)
Other services^a^		378	187 (58**–**418)		377	191 (57**–**502)		264	127 (0**–**391)		303	153 (0**–**420)
Total Costs		2178	1027 (614–3084)		1302	690 (376–1182)		944	568 (308–955)		1087	607 (329–1053)
Second Year Post Discharge	13			288			560			861		
Outpatient sector		433	234 (176**–**530)		372	218 (110**–**474)		313	224 (105**–**406)		335	224 (107**–**421)
Inpatient sector		661	0 (0**–**607)		318	0 (0**–**0)		181	0 (0**–**0)		234	0 (0**–**0)
Prescribed medications		1517	53 (26**–**92)		311	36 (0**–**116)		156	30 (0**–**79)		228	32 (0**–**92)
Other services^a^		20	0 (0**–**0)		185	0 (0**–**76)		83	0 (0**–**56)		116	0 (0**–**58)
Total Costs		2631	530 (229–1282)		1185	331 (142–994)		732	332 (130–687)		912	332 (134–751)
Third Year Post Discharge	6			174			304			484		
Outpatient sector		386	417 (216**–**520)		395	265 (146**–**497)		363	259 (138**–**461)		375	263 (140**–**481)
Inpatient sector		1746	0 (0**–**3412)		478	0 (0**–**0)		240	0 (0**–**0)		344	0 (0**–**0)
Prescribed medications		93	83 (32**–**150)		367	47 (14**–**125)		225	44 (12**–**100)		275	45 (12**–**107)
Other services^a^		164	44 (0**–**173)		172	0 (0**–**85)		99	0 (0**–**60)		126	0 (0**–**77)
Total Costs		2389	1068 (652–3575)		1412	389 (188–855)		928	385 (181–845)		1120	388 (183–867)

^a^Note that other services include: Remedies (e.g., physiotherapy, massage, manual therapy, occupational therapy); Medical devices (e.g., measurement devices, medical devices, inhalation devices); Midwife services (e.g., antenatal preparation, home visits after birth); Driving services (e.g., ambulance service, patient transportation); Other (e.g., household help, home care necessary for medical treatment);

Abbreviations: *GA* gestational age, *IQR* interquartile range, *PTB* preterm birth, *PTL* preterm labour

## Discussion

Available economic data on PTL/PTB mainly focuses on infants; to our knowledge, this was the first study to assess maternal resource use and costs among PTL/PTB mothers in Germany. We found median maternal costs incurred by PTL/PTB mothers were €2130 during pregnancy, €2037 during the delivery hospitalisation, and €607, €332, and €388 in the first, second, and third years, respectively, after delivery. Our study revealed variations in resource use, LOS, and costs according to GA at delivery, with higher estimates of resource use and costs observed in PTL/PTB mothers with lower GA infants at birth. These differences were observed during delivery hospitalisation and persisted after delivery. Trends were similar when considering mean and median costs.

Previously published studies, although using different methodologies, have shown a strong inverse relationship between infant costs associated with PTB and GA [13, 14, 28–31]. In our study, differences in cost according to GA were most marked during the delivery hospitalisation and during the first year after delivery. This is in agreement with previous studies, which have found that differences in direct costs associated with care for the infant according to GA are less marked for the second and third year after delivery [32]. In terms of maternal costs, the published evidence is limited. A recent analysis by Steetskamp et al., which examined data from a single German hospital in Mainz, found average costs of €332 per day and an average LOS of 13.5 days for mothers who gave birth to a PTB infant; taken together, these result in average maternal costs of €4482 per delivery hospitalisation [33]. An analysis of Swedish registry data from 2006 reported a mean LOS of between three and nine days for mothers, depending on the GA of the infant, and associated mean maternal costs ranged from €3167–€5173, depending on the GA [13]. Comparing costs across studies is complex, as differences in the setting, study population, timeframe, and the costs of resources in different countries make it difficult to generalise results [9]. Nonetheless, previous studies have shown a strong correlation between GA, LOS, and maternal costs, which is similar to the trends we describe here [13]. In our study, the high costs observed within the lowest GA group appeared to persist after delivery, although it should be noted that our estimates were based on small numbers. Our study was not designed to assess the potential causes of such persistently increasing costs after delivery. Although there are possible causal mechanisms that could explain these higher costs, for example, increased care needs arising because of poor mental health caused by giving birth to a very pre-term infant, [20, 21] it is also possible that the same factors that caused mothers to experience very preterm birth independently had an impact on resource use and costs. Such risk factors include underlying chronic medical conditions (e.g., diabetes, hypertension, depression [34]) as well as social factors such as socioeconomic status [35].

### Strengths and limitations

This analysis was subject to several limitations. First, as this was a secondary analysis of administrative insurance claims data not collected for research purposes, the availability of some variables was limited. The lack of data on the exact number of physician visits meant that we were restricted to estimating the resource use of ‘at least one physician visit per quarter’ rather than the true number of physician visits. We were also limited by the lack of detailed medical history (such as availability of more specific GAs). To maximise the sample and increase the generalisability of results, we reduced the length of minimum medical history required for inclusion to nine months prior to delivery; this meant we were unable to characterise the obstetric history of women in detail. In addition, we allowed mothers to enter the cohort until the end of the study period, with the trade-off that we would not have long-term follow-up data for women entering toward the end of the study period. While these strategies allowed us to maximise the starting cohort sample size, the sample size decreased with time, as only mothers with recorded deliveries prior to 1 January 2011 (38%) could be potentially observed in the database for the entire three years of follow-up. Although the potential impact of loss to follow-up should be born in mind when interpreting the results, we do not expect significant bias to be introduced, as the main cause of the reduction in the sample size over time was women entering the study close to the end of the study period. The resulting lack of precision and wide CIs, however, is a limitation. A further limitation is the lack of an ability to link data between mothers and infants, which could lead us to miss instances of PTB, and prevented any assessment of combined mother-infant costs. As with any administrative database study, missing or potentially erroneously recorded diagnostic codes is also a limitation.

Our manuscript aimed to assess the total costs associated with all PTL/PTB mothers. However, it is likely that mothers of multiples or mothers with complications during birth have greater resource use and costs compared with mothers of singletons without complications. Further research on how resource use and costs vary not only according to GA, but to the parity and medical history of the mothers, would be of value. In addition, although our aim was to provide a descriptive overview of costs associated with PTL/PTB in Germany, future studies utilising an appropriately constructed control group of non-PTL mothers who deliver at term—to quantify the excess costs associated with PTL/PTB—would also be of interest. We found no published cost estimates of pregnancies leading to term birth in Germany to contrast our estimates of costs associated with PTL/PTB; however, prior comparative studies have found that mothers who experience PTL/PTB have significantly worse health outcomes, [17] longer LOS, [13] and higher resource use [18] compared with mothers who do not experience PTL/PTB, which would likely indicate excess associated costs compared with non-PTL mothers who deliver at term. We were not able to assess the total costs for all PTL/PTB mothers, including costs incurred by their infants, as it was not possible to link all mothers to infants in the AOK database. However, previous studies in the German setting [12] and other countries [8, 36, 37] have found that PTB infants have higher resource use and costs compared with term infants, and that infant costs are generally higher than costs incurred by mothers [13]. This indicates that, had we been able to link all mothers to their infants, the estimated total costs associated with PTL/PTB among mothers and their infants would have been considerably higher. Our analyses were also limited to an assessment of direct costs. It is important to note that PTL/PTB also results in indirect costs, such as earnings lost from taking time off work or costs associated with travel to and from the hospital [30]. It should be noted that costs reflected those incurred within the respective years from 2009 to 2013. Although this may pose a limitation, the consumer price index for healthcare in Germany according to Eurostat [38] during 2009–2013 showed an average annual increase of 1.5%; therefore, the effect of year-on-year changes during our study period was small.

Despite these limitations, our analysis provides up-to-date data on the prevalence and maternal PTL/PTB costs and resource use in a country where limited information on these exist. The apparent trend of declining costs with increasing GA we observed is interesting, and highlights the importance of considering maternal costs, in addition to infant costs, in burden of illness studies of PTL/PTB. Future studies investigating the causes of the increase in costs at lower GAs, particularly over the longer term, would be of great interest. Our analysis is also strengthened by reflecting direct medical costs from the third-party payer perspective, and a long follow-up, relative to previously published studies.

## Conclusions

Our findings suggest that estimates of national costs of PTL/PTB should take the contribution of maternal short and long-term costs into account. Although analyses by GA were descriptive, costs appeared to be greater for mothers who delivered infants at lower GAs. This gradient suggests that PTL/PTB may also have an impact on maternal resource use and costs. Further studies utilising appropriate control groups and methods to control for confounding are needed to adequately assess this. Although data on absolute costs cannot determine the most efficient allocation of resources alone, [30] our findings will be of use for policy makers responsible for planning the delivery of maternal services in Germany, as well as to researchers developing cost-effectiveness models. Further research is required to fully understand the underlying causes of the resource use and costs that are observed over multiple years for mothers diagnosed with PTL/PTB.

## Additional files

Additional file 1: **Table S1.** Drg and ops codes used to identify deliveries. Diagnosis-related group and operationen- und prozedurenschlüssel codes used while examining the statutory health insurance (SHI) sample of aok hessen (versichertenstichprobe AOK Hessen/KV Hessen) to identify deliveries. (DOCX 21 kb)

Additional file 2: **Table S2.** Codes used to identify ptl/ptb deliveries. International classification of diseases, 10th revision and diagnosis-related group and operationen- und prozedurenschlüssel codes used while examining the statutory health insurance (SHI) sample of aok hessen (versichertenstichprobe AOK Hessen/KV Hessen) to identify preterm birth/preterm labour deliveries. (DOCX 15 kb)

## Abbreviations

CCI: Charlson Comorbidity Index
CI: Confidence interval
DRG: Diagnosis-related group
ECG: Echocardiogram
GA: Gestational age
GP: General practitioner
ICD-10: International Classification of Diseases, 10th Revision
ICU: Intensive care unit
IQR: Interquartile range
IQWiG: Institute for Quality and Efficiency in Health Care
LBW: Low-birth-weight
LOS: Length of stay
LTFU: Lost-to-follow-up
MRI: Magnetic resonance imaging
OPS: Operationen- und Prozedurenschlüssel
PTB: Preterm birth
PTL: Preterm labour
SHI: Statutory Health Insurance
US: United States

## Footnotes

1 Linkage between mothers and infants in AOK Hessen is not possible if the mother or the infant (or both) are not insured with the AOK, or if the mother or infant have not been included in the randomly selected SHI sample. This meant that, in total, linkage to infants was only possible for 16.3% of all mothers in the dataset.
