# Supplementary material for: Short and long-term costs among women experiencing preterm labour or preterm birth: the German experience
Source: BMC Pregnancy Childbirth. 2018 Jul 4;18:284. doi: 10.1186/s12884-018-1912-0 (PMC6030749; doi:10.1186/s12884-018-1912-0)
Supplement: Supplementary file 1 — Table S1. Drg and ops codes used to identify deliveries. Diagnosis-related group and operationen- und prozedurenschlüssel codes used while examining the statutory health insurance (SHI) sample of aok hessen (versichertenstichprobe AOK Hessen/KV Hessen) to identify deliveries. (DOCX 21 kb) [file 12884_2018_1912_MOESM1_ESM.docx]

**Table S1. DRG and OPS Codes Used to Identify Deliveries**

| **DRG** | **Description** |
| --- | --- |
| O02A, O02B, O60A, O60B, O60C, O60D | Vaginal delivery |
| O01A, O01B, O01C, O01D, O01E, O01F, O01G, O01H | Cesarean section |
| **OPS** | ***Description*** |
| 5--72 | Breech and instrumental delivery |
| 5-73 | Other operations for induction of labour and birth |
| 5-740 | Classical cesarean section |
| 5-741 | Cervical cesarean section |
| 5-742 | Extraperitoneal cesarean section |
| 5-745 | Sectio cesarea combined with other gynaecological operations |
| 5-749 | Cesarean section, not otherwise specified |
| 5-759 | Other obstetric operations |
| 9-260 | Routine supervision of normal delivery |
| 9-261 | Routine supervision of risc delivery |
| 9-268 | Routine supervision of delivery, not elsewhere classified |

Abbreviations: DRG = diagnosis-related group; OPS = Operationen- und ProzedurenSchlüssel
