# Supplementary material for: Short and long-term costs among women experiencing preterm labour or preterm birth: the German experience
Source: BMC Pregnancy Childbirth. 2018 Jul 4;18:284. doi: 10.1186/s12884-018-1912-0 (PMC6030749; doi:10.1186/s12884-018-1912-0)
Supplement: Supplementary file 2 — Table S2. Codes used to identify ptl/ptb deliveries. International classification of diseases, 10th revision and diagnosis-related group and operationen- und prozedurenschlüssel codes used while examining the statutory health insurance (SHI) sample of aok hessen (versichertenstichprobe AOK Hessen/KV Hessen) to identify preterm birth/preterm labour deliveries. (DOCX 15 kb) [file 12884_2018_1912_MOESM2_ESM.docx]

**Table S2. ICD-10 and DRG Codes Used to Identify PTL/PTB Deliveries**

| **ICD-10** | **Description** |
| --- | --- |
| O60.0 | Preterm labor without delivery |
| O60.1 | Preterm spontaneous labor with preterm delivery |
| O60.2 | Preterm spontaneous labor with term delivery |
| P05. 0 | Newborn light for gestational age |
| P05.1 | Newborn small for gestational age |
| P07.0 | Newborn with extremely low birth weight, Birth weight 999 g or less |
| P07.00 | Newborn with extremely low birth weight, birth weight 499 g or less |
| P07.01 | Newborn with extremely low birth weight, birth weight 500-749 g |
| P07.02 | Newborn with extremely low birth weight, birth weight 750-999 g |
| P07.1 | Newborn with other low birth weight, birth weight 1000-2499 g |
| P07.10 | Newborn with other low birth weight, birth weight 1000-1249 g |
| P07.11 | Newborn with other low birth weight, birth weight 1250-1499 g |
| P07.12 | Newborn with other low birth weight, birth weight 1500-2499 g |
| P07.2 | Newborn with extreme immaturity |
| P07.3 | Other preterm infants |
| **DRG** | **Description** |
| P61A | Newborn, AdmWt < 600 g with significant OR-procedure |
| P61B | Newborn, AdmWt < 600 g without significant OR-procedure |
| P61C | Newborn, AdmWt 600-749 g with significant OR-procedure |
| P61D | Newborn, AdmWt 600-749 g without significant OR-procedure |
| P61E | Newborn, AdmWt < 750 g, died < 29 days after admission |
| P62 A | Newborn, AdmWt 750-874 g with significant OR-procedure |
| P62B | Newborn, AdmWt 750-874 g without significant OR-procedure |
| P62C | Newborn, AdmWt 875-999 g without significant OR-procedure |
| P62D | Newborn, AdmWt 875-999 g, died < 29 days after admission |
| P03A | Newborn, AdmWt 1000-1499 g with significant OR-procedure or  artificial respiration > 95 hours, or with multiple severe problems and artificial respiration > 479 hours |
| P03B | Newborn, AdmWt 1000-1499 g with significant OR-procedure or artificial respiration > 95 hours with multiple severe problems and artificial respiration > 120 and < 480 hours |
| P03C | Newborn, AdmWt 1000-1499 g with significant OR-procedure or  artificial respiration > 95 hours, or artificial respiration > 120 hours without multiple severe problems; without long-term complex OR- procedures |
| P63Z | Newborn, AdmWt 1000-1249 g without significant OR-procedure, without artificial respiration > 95 hours |
| P64Z | Newborn, AdmWt 1250-1499 g without significant OR-procedure, without artificial respiration > 95 hours |
| P04A | Newborn, AdmWt 1500-1999 g with significant OR-procedure or artificial respiration > 95 hours with multiple severe problems, with artificial respiration >40 hours |
| P04B | Newborn, AdmWt 1500-1999 g with significant OR-procedure or artificial respiration > 95 hours with multiple severe problems, without artificial respiration > 240 hours |
| P04C | PTB Newborn, AdmWt 1500-1999 g with significant OR-procedure or artificial respiration > 95 hours with multiple severe problems, with artificial respiration > 240 hours |
| P65A | PTB Newborn, AdmWt 1500-1999 g without significant OR-procedure, without artificial respiration > 95 hours, with multiple severe problems |
| P65B | Newborn, AdmWt 1500-1999 g without significant OR-procedure, without artificial respiration > 95 hours, with severe problem |
| P65C | Newborn, AdmWt 1500-1999 g without significant OR-procedure, without artificial respiration > 95 hours, with other problem |
| **DRG** | **Description** |
| P65D | Newborn, AdmWt 1500-1999 g without significant OR-procedure, without artificial respiration > 95 hours, without problem |
| P05A | Newborn, AdmWt 2000-2499 g with significant OR-procedure or artificial respiration > 95 hours with multiple severe problems |
| P05B | Newborn, AdmWt 2000-2499 g with significant OR-procedure or artificial respiration > 95 hours with multiple severe problems, without artificial respiration > 120 hours |
| P05C | Newborn, AdmWt 2000-2499 g with significant OR-procedure or artificial respiration > 95 hours without multiple severe problems |
| P66B | Newborn, AdmWt 2000-2499 g without significant OR-procedure, without artificial respiration > 95 hours, with severe problem |
| P66C | Newborn, AdmWt 2000-2499 g without significant OR-procedure, without artificial respiration > 95 hours, with other problem |

Abbreviations: DRG = diagnosis-related group; ICD-10 = International Classification of Diseases, 10th revision; PTB = preterm birth; PTL = preterm labour
